# Policy gradient rules for populations of spiking neurons

**DOI:** 10.1186/1471-2202-12-S1-P111

**Published:** 2011-07-18

**Authors:** Johannes Friedrich, Robert Urbanczik, Walter Senn

**Affiliations:** 1 Department of Physiology, University Bern, Bern, Switzerland

## 

Population coding is widely regarded as a key mechanism for achieving reliable behavioral decisions given a high neuronal variability. Here, we present two general recipes to derive learning rules from a policy gradient approach for different neural codes and decision making networks, one based on partial integration across feature values, and one based on linear approximation around a target feature. The first technique leads to a tightly code-specific learning rule where details of the code-irrelevant spiking information are integrated away and the code-specificity enters at the synaptic level. The second technique yields modular learning rules which can be weakly code-specific, with a spike-timing dependent base synaptic plasticity rule which is modulated by a code specific population and decision signal. Decisions can be binary, multi-valued, or even continuous-valued. For illustration, we consider a spike count and a spike latency code. We test them on simple model problems and assess the superiority of tight over weak code-specificity with respect to the performance. While code-specific rules increase the performance only marginally when considering a single neuron [1], our tightly code-specific rule designed for population coding can strongly boost performance. Both code-specific learning rules improve in performance with increasing population size as opposed to standard reinforcement learning [2]. For mathematical clarity we presented the rules for an episodic learning scenario. But a biological plausible implementation of a fully online scheme is also possible [2,3].
